# Serum pyruvate and lactate predict immunotherapy efficacy in advanced gastric cancer: a prospective biomarker study

**DOI:** 10.3389/fimmu.2025.1663362

**Published:** 2025-09-17

**Authors:** Chang Liu, Ying Dai, Jiru Wang, Jingjing Wu, Bin Wei

**Affiliations:** ^1^ Department of Oncology, The Affiliated Huaian No.1 People’s Hospital of Nanjing Medical University, Huai’an, China; ^2^ Department of stomatology, The Affiliated Huaian No.1 People’s Hospital of Nanjing Medical University, Huai’an, China; ^3^ Department of Hematology, The Affiliated Huaian No.1 People’s Hospital of Nanjing Medical University, Huai’an, China; ^4^ Northern Jiangsu Institute of Clinical Medicine, Nanjing Medical University, Huai’an, China; ^5^ Department of Oncology, The Huaian Clinical College of Xuzhou Medical University, Huai’an, China

**Keywords:** glycolytic metabolites, pyruvate, lactate, immunotherapy efficacy, gastric cancer, prospective study

## Abstract

**Background:**

While immunotherapy has redefined clinical paradigms for advanced gastric cancer, reliable efficacy prediction remains a critical unmet challenge. Unlike invasive tissue-based predictors, circulating biomarkers offer non-invasive monitoring potential. This study investigated serum energy metabolites, whose dysregulation drove immune evasion, as predictors of therapeutic efficacy in advanced gastric cancer receiving first-line chemoimmunotherapy.

**Methods:**

We conducted a prospective observational study involving 52 patients with advanced gastric cancer receiving first-line chemoimmunotherapy. Serum metabolites of glycolysis and tricarboxylic acid (TCA) cycle were quantified via high-performance liquid chromatography-tandem mass spectrometry. Therapeutic response, progression-free survival (PFS), and overall survival (OS) were served as evaluation endpoints.

**Results:**

Patients exhibiting decreased serum concentrations of glycolytic metabolites (lactate and pyruvate) demonstrated significantly higher disease control rate (DCR) compared to those with elevated concentrations. Elevated serum lactate and pyruvate were significantly associated with inferior PFS and OS. Multivariate Cox regression established low lactate and pyruvate as independent prognostic factors for improved PFS and OS. However, no significant associations were observed between serum TCA cycle metabolites (citrate, isocitrate, α-ketoglutarate, succinate, fumarate, malate, and oxaloacetate) and DCR, PFS, or OS.

**Conclusion:**

Our findings suggest that serum lactate and pyruvate as non-invasive glycolytic biomarkers with substantial predictive value for immunotherapy efficacy in advanced gastric cancer, requiring validation in larger cohorts to guide therapeutic decisions.

## Introduction

Gastric cancer remains a significant global health burden, ranking as the fifth most diagnosed and fifth deadliest malignancy worldwide (1). Traditional treatment modalities, including surgical resection, chemotherapy, and radiotherapy, often show limited efficacy in the advanced stages of the disease (2). Immunotherapy has emerged as a cornerstone therapeutic strategy in gastric cancer, demonstrating significant survival improvements (3). The approved tissue-based biomarkers for gastric cancer immunotherapy include programmed cell death 1 ligand 1 (PD-L1), microsatellite status, and tumor mutational burden (4). However, tissue-based biomarkers capture only a single spatiotemporal snapshot and fail to reflect intra-tumoral and inter-tumoral heterogeneity across timepoints (5). Liquid biopsy is increasingly utilized in precision oncology, encompassing five key modalities: circulating tumor DNA, circulating tumor cells, exosomes, extracellular RNAs, and metabolic signatures. These modalities collectively enable non-invasive disease monitoring, therapeutic guidance, and dynamic response assessment (6). Consequently, identifying robust liquid biopsy biomarkers to guide immunotherapy in gastric cancer is clinically essential.

Energy metabolism sustains physiological functions by converting nutrients into adenosine triphosphate (ATP) for cellular homeostasis (7). By serving as a fundamental energy source via tricarboxylic acid (TCA) and glycolytic pathways, glucose metabolism delivers both ATP for cellular and organismal growth, and biosynthetic precursors for macromolecular assembly (8). Substantial evidence demonstrates that during malignancy, glycometabolites derived from both pathways collectively orchestrate tumor immunomodulation, mechanistically exemplified by: (i) tumor-derived lactate generated via glycolytic pyruvate conversion acidifying the microenvironment to compromise anti-tumor immunity; while (ii) TCA cycle metabolites, such as α-ketoglutarate, succinate, and fumarate, exerting diverse immunomodulatory effects on immune cell subsets, such as cytotoxic T cells, macrophages, and regulatory T cells (7, 9). Although tumor cells retain the energy metabolism of TCA cycle, they undergo metabolic reprogramming and transform into a metabolic feature dominated by glycolysis, which is known as the Warburg effect (10). And numerous studies have shown that targeting energy metabolism may become a potential strategy for enhancing the efficacy of anti-tumor treatments, including immunotherapy (11). Therefore, the metabolites related to the TCA cycle and glycolysis have the potential to serve as biomarkers for predicting immunotherapy efficacy in malignancies.

Metabolomics leverages high-throughput technologies to profile metabolites in biofluids, and tissues, profoundly advancing our understanding of malignancies. This approach is critical for tumor biomarker discovery and enables real-time prediction of tumor burden and treatment response (12, 13). Here, we employed targeted metabolomics to quantitatively profile serum energy metabolites, investigated their associations with clinical efficacy, and evaluated their potential to predict survival outcomes in advanced gastric cancer patients receiving first-line chemoimmunotherapy.

## Patients and methods

### Study design

This prospective observational cohort study was conducted in the Department of Oncology at the Affiliated Huaian No.1 People’s Hospital of Nanjing Medical University from June 2021 to December 2024. It aimed to investigate associations between serum metabolites involved in glycolysis and TCA cycle and both clinical response and prognosis in advanced gastric cancer patients receiving first-line chemoimmunotherapy. The study protocol was approved by the Human Ethics Committee of the Affiliated Huaian No.1 People’s Hospital of Nanjing Medical University (YX-2021-058-01). All patients signed their informed consent.

### Study population

The study population included 52 patients who were diagnosed with advanced gastric cancer at the Affiliated Huaian No.1 People’s Hospital of Nanjing Medical University. Inclusion criteria were as follows: Age >18 years old; untreated, unresectable locally advanced or metastatic gastric cancer; human epidermal growth factor receptor 2 (HER-2) status (negative or low expression) confirmed by immunohistochemistry and/or fluorescence *in situ* hybridization; measurable disease according the Response Evaluation Criteria in Solid Tumors (RECIST); Eastern Cooperative Oncology Group (ECOG) performance status of 0 or 1. Exclusion Criteria were as follows: Prior immunotherapy; active autoimmune diseases requiring immunosuppression, uncontrolled infections, or untreated central nervous system metastases; history of other malignancies within 5 years; severe hypersensitivity and major surgery within 4 weeks. The patient demographics, major metastatic lesion, grade of differentiation, status of HER-2 and PD-L1 combined positive score (CPS), etc. were all collected.

### Therapeutic regimen

All eligible patients received first-line anti-PD-1 therapy combined with chemotherapy. The standard chemotherapy regimens include fluoropyrimidines (fluorouracil, capecitabine or S1) plus platinum agents (oxaliplatin or cisplatin), alongside anti-PD-1 drugs (nivolumab, sintilimab, or tislelizumab), which were administered every 3 weeks until disease progression or intolerable toxicity.

### Outcome evaluation

Quantification of tumor burden was performed through computed tomography and/or magnetic resonance imaging at baseline. Subsequent therapeutic response monitoring was conducted at protocol-defined intervals, specifically every 2 to 3 treatment cycles, using serial radiographic evaluations. Therapeutic responses were categorized as complete response (CR), partial response (PR), stable disease (SD) and progressive disease (PD) according to the RECIST criteria. Survival endpoints were quantified as follows: overall survival (OS) spanned from first-line therapeutic initiation to mortality or censoring at last documentation, while progression-free survival (PFS) encompassed the interval between treatment commencement and either disease progression or death from any cause.

### Serum sample preparation

The serum samples were thawed at 4 °C, mixed with the isotope internal standard substance, and then cold methanol/acetonitrile solution was added. Subsequently, the samples were subjected to thorough vortexing, ultrasonic treatment at low temperature, and protein precipitation at -20 °C. Afterwards, the supernatants were collected by centrifugation and dried in a vacuum centrifuge. Finally, the samples were re-dissolved in an acetonitrile/water mixture, thoroughly shaken, then centrifuged to collect the supernatant, which was used for high performance liquid chromatography-tandem mass spectrometry (HPLC-MS/MS) analysis.

### HPLC-MS/MS analysis

Analyses were performed using an ultra-HPLC (1290 Infinity LC, Agilent Technologies) coupled to a QTRAP 6500+ (AB Sciex). The mobile phase consisted of two components: (A) 50 mM ammonium acetate in water with 1.2% ammonium hydroxide, and (B) 1% acetylacetone in acetonitrile. Samples were maintained at 4 °C in the autosampler, while the column temperature was controlled at 35 °C. Chromatographic separation was carried out using a gradient elution method at a flow rate of 300 μl/min, with a precise injection volume of 2 μL per sample. The quality control samples were used to assess system stability and repeatability, while a standard metabolite mixture was employed for chromatographic retention time calibration. Ion pairs were detected via multiple reaction monitoring under negative electrospray ionization conditions. Chromatographic peaks were integrated using MultiQuant software. Glycometabolites involved in glycolysis and TCA cycle were identified by retention time alignment with authenticated standards and quantified via isotope dilution mass spectrometry using stable isotope-labeled internal standards.

### Statistical analysis

All statistical analyses were conducted using the Statistical Package for the Social Sciences (SPSS) software. Independent samples t-tests, which selected based on Levene’s test for equality of variances, were applied to examine metabolite associations with clinical parameters. Metabolites levels were assessed for normality via the Shapiro-Wilk test, skewed data underwent log-transformation, with the mean serving as the threshold for grouping into low- and high-expression groups. The association between metabolites expression and therapeutic response was evaluated by Chi-square test or Fisher’s exact tests, and the odds ratio (OR) values and 95% confidence intervals (95% CIs) were calculated. PFS, and OS distributions were analyzed through Kaplan-Meier curves and Log-rank tests. Time-dependent receiver operating characteristic (ROC) curves with area under the curve (AUC) values and 95% CIs were employed to analyze the predictive value of these metabolites for 6-month PFS and 12-month OS. Univariate and multivariate Cox regression analyses with hazard ratios (HR) and 95% CI were employed to determine the independent predictors of survival. *P* value < 0.05 was considered statistically significant.

## Results

### Patient characteristics

**Figure 1:**
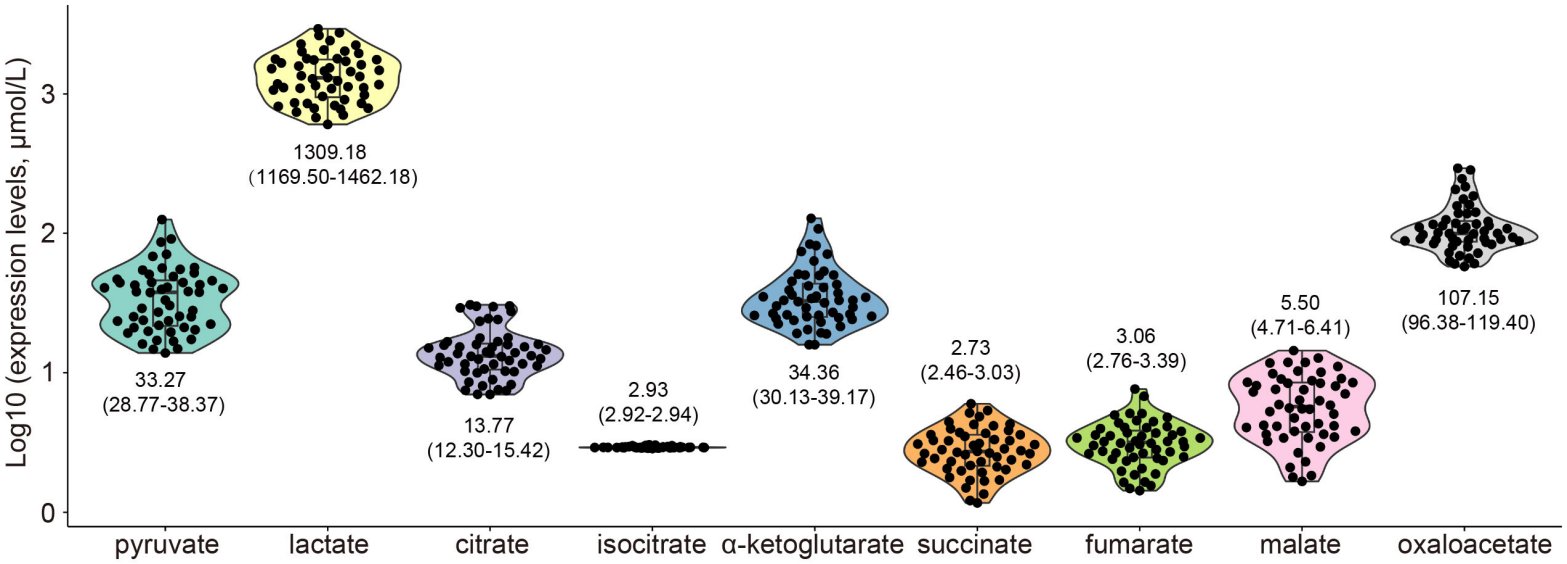
The expression of serum glycometabolites in patients with advanced gastric cancer. The graph illustrated the expression of glycometabolites, expressed as the mean with 95% CI, including pyruvate, lactate, citrate, isocitrate, α-ketoglutarate, succinate, fumarate, malate, and oxaloacetate.

This study enrolled 52 advanced gastric cancer patients (44 males and 8 females) with a median age of 61 years (range: 47-80 years). The major metastatic lesions among these patients comprised hepatic metastases (19 cases), non-regional lymph node metastases (17 cases), peritoneal metastases (12 cases), pulmonary metastases (3 cases), and splenic metastasis (1 case). All patients exhibited proficient mismatch repair, with 26 HER2-negative (0) and 26 HER2-low (1+/2+). The PD-L1 CPS distribution showed 18 patients ≥ 5 and 34 patients < 5. Two glycolytic metabolites and seven TCA cycle metabolites were quantified in all patients, with the following mean concentrations and 95% CI (μmol/L): pyruvate: 33.27 (28.77-38.37), lactate: 1309.18 (1169.50-1462.18), citrate: 13.77 (12.30-15.42), isocitrate: 2.93 (2.92-2.94), α-ketoglutarate: 34.36 (30.13-39.17), succinate: 2.73 (2.46-3.03), fumarate: 3.06 (2.76-3.39), malate: 5.50 (4.71-6.41), oxaloacetate: 107.15 (96.38-119.40). All data are presented in **Table 1**, **Figure 1**. The analysis of therapeutic response demonstrated an objective response rate (ORR) of 26.9% (14/52) and a disease control rate (DCR) of 76.9% (40/52). The median PFS was 7.6 months (95% CI: 6.5-8.7) without censoring events, and the median OS was 12.6 months (95% CI: 9.6-15.6) with 6 patients censored.

**Table 1:** Association between serum glycometabolites and clinicopathologic variables of advanced gastric cancer.

Clinical variables	No.	Metabolite expression (Mean, 95%CI)								
		Pyruvate	Lactate	Citrate	Isocitrate	α-Ketoglutarate	Succinate	Fumarate	Malate	Oxaloacetate
Age, years										
<65	30	35.5 (28.9-42.0)	1411.9 (1205.5-1618.3)	16.2 (13.5-19.0)	2.94 (2.92-2.95)	38.9 (30.5-47.3)	3.0 (2.56-3.34)	3.2 (2.8-3.7)	6.5 (5.4-7.6)	115.9 (96.3-135.4)
≥65	22	41.6 (29.8-53.3)	1418.9 (1147.6-1690.2)	13.3 (10.9-15.7)	2.92 (2.91-2.94)	38.8 (28.4-49.2)	2.9 (2.37-3.40)	3.3 (2.7-4.0)	6.1 (4.5-7.7)	116.6 (92.2-141.1)
* P* value		0.322	0.966	0.115	0.196	0.982	0.831	0.784	0.621	0.960
Gender										
Males	44	39.3 (32.4-46.1)	1376.4 (1208.4-1544.4)	15.5 (13.4-17.6)	2.93 (2.92-2.94)	38.0 (31.6-44.4)	2.93 (2.60-3.26)	3.2 (2.9-3.6)	6.3 (5.3-7.3)	117.6 (100.4-134.7)
Females	8	31.5 (16.7-46.3)	1626.5 (1064.3-2188.8)	12.3 (8.1-16.6)	2.91 (2.89-2.94)	43.6 (17.0-70.2)	2.88 (1.86-3.91)	3.6 (2.0-5.1)	6.7 (4.0-9.3)	108.6 (84.9-132.3)
		0.358	0.260	0.220	0.338	0.528	0.990	0.458	0.754	0.665
G stage										
G1-2	10	30.2 (19.3-42.1)	1453.5 (969.3-1937.7)	13.4 (11.4-15.4)	2.92 (2.90-2.93)	37.8 (24.4-51.2)	3.07 (2.01-4.13)	3.1 (2.3-3.9)	6.8 (4.2-9.4)	109.3 (61.8-156.9)
G3	42	39.9 (32.6-47.2)	1405.7 (1233.0-1578.3)	15.4 (13.1-17.6)	2.93 (2.92-2.95)	39.1 (31.8-46.5)	2.88 (2.58-3.20)	3.3 (2.9-3.7)	6.2 (5.2-7.2)	117.8 (102.2-133.4)
* P* value		0.205	0.815	0.412	0.376	0.867	0.646	0.643	0.621	0.653
HER-2										
0	26	41.1 (32.0-50.1)	1492.8 (1240.5-1745.1)	15.4 (12.8-17.9)	2.93 (2.91-2.95)	43.1 (31.8-54.4)	3.1 (2.6-3.6)	3.5 (3.0-4.1)	6.7 (5.3-8.1)	118.7 (99.9-137.4)
1-2	26	35.1 (26.5-43.6)	1336.9 (1128.5-1545.3)	14.6 (11.7-17.5)	2.93 (2.91-2.94)	34.7 (28.5-40.9)	2.7 (2.4-3.1)	3.0 (2.6-3.5)	6.0 (4.8-7.2)	113.7 (89.6-137.8)
* P* value		0.325	0.331	0.677	0.409	0.182	0.240	0.157	0.441	0.741
PD-L1 CPS										
<5	34	38.0 (29.7-46.4)	1464.8 (1272.5-1655.1)	16.0 (13.3-18.7)	2.93 (2.92-2.95)	39.7 (30.9-48.5)	3.0 (2.6-3.4)	3.5 (3.0-4.0)	6.6 (5.5-7.8)	122.7 (103.8-141.5)
≥5	18	38.1 (29.4-46.9)	1322.4 (1013.3-1631.5)	13.1 (11.5-14.7)	2.92 (2.91-2.93)	37.3 (28.6-46.0)	2.8 (2.4-3.2)	2.8 (2.3-3.3)	5.8 (4.2-7.3)	104.0 (78.9-129.0)
* P* value		0.986	0.402	0.063	0.096	0.715	0.592	0.062	0.368	0.229

### Association between serum glycometabolites and clinicopathological parameters

Here, we examined serum glycometabolite associations with clinicopathological parameters. Statistical analysis revealed no significant associations (all *P* > 0.05) between any of the nine metabolites (pyruvate, lactate, citrate, isocitrate, α-ketoglutarate, succinate, fumarate, malate, and oxaloacetate) and clinicopathological parameters including age, sex, histological differentiation grade, HER2 status, or PD-L1 CPS (**Table 1**).

### Relationship between serum glycometabolites and clinical efficacy

Among 52 evaluable patients, therapeutic responses comprised PR in 14 cases (26.9%), SD in 26 (50.0%), and PD in 12 (23.1%) per the RECIST criteria. Patients were stratified into low- and high-expression groups using the mean of log-transformed serum metabolite concentrations as the grouping threshold. No statistically significant differences in ORR were observed across subgroups stratified by nine different metabolites (all *P* > 0.05, **Table 2**). Patients with low levels of glycolytic metabolites (pyruvate: 96.0% *vs.* 59.3%, *P* = 0.002; lactate: 92.3% *vs.* 61.5%, *P* = 0.019) exhibited significantly higher DCR compared to respective high-level groups (**Table 2**). TCA cycle metabolites, including α-ketoglutarate (90.3% vs. 57.1%, *P* = 0.008, **Table 2**), and fumarate (91.7% vs. 64.3%, *P* = 0.024; **Table 2**), demonstrated significant associations with DCR, while citrate, isocitrate, succinate, malate, and oxaloacetate exhibited no statistical linkage to DCR (all *P* > 0.05, **Table 2**). Besides, patients with PD-L1 CPS ≥5 demonstrated markedly improved ORR (44.6% *vs.* 17.6%, *P* = 0.038) and DCR (100% *vs.* 64.7%, *P* = 0.004) compared to those with PD-L1 CPS < 5 (**Table 2**).

**Table 2:** Association of serum glycometabolites with ORR and DCR in advanced gastric cancer treated with chemoimmunotherapy.

Clinical efficacy	Metabolite expression																			
	Pyruvate		Lactate		Citrate		Isocitrate		α-Ketoglutarate		Succinate		Fumarate		Malate		Oxaloacetate		PDL1 CPS	
	Low	High	Low	High	Low	High	Low	High	Low	High	Low	High	Low	High	Low	High	Low	High	<5	≥5
No.	25	27	26	26	28	24	31	21	31	21	26	26	24	28	25	27	30	22	34	18
ORR																				
PR	8	6	9	5	7	7	7	7	8	6	7	7	9	5	8	6	8	6	6	8
SD+PD	17	21	17	21	21	17	24	14	23	15	19	19	15	23	17	21	22	16	28	10
* P* value	0.427		0.211		0.736		0.391		0.825		1.000		0.130		0.427		0.961		0.038	
OR (95% CI)	0.61 (0.18-2.09)		0.45 (0.13-1.60)		1.24 (0.36-4.22)		1.17 (0.50-5.91)		1.15 (0.33-3.98)		1.00 (0.29-3.41)		0.36 (0.10-1.29)		0.61 (0.18-2.09)		1.03 (0.30-3.56)		3.73 (1.04-13.45)	
DCR																				
PR+SD	24	16	24	16	21	19	24	16	28	12	21	18	22	18	24	18	24	16	22	18
PD	1	11	2	10	7	5	7	5	3	9	4	8	2	10	3	9	6	6	12	0
* P* value	0.002		0.019		0.754		0.918		0.008		0.324		0.024		0.099		0.740		0.004	
OR (95% CI)	0.06 (0.01-0.52)		0.13 (0.03-0.69)		1.27 (0.34-4.67)		0.93 (0.25-3.46)		0.14 (0.33-0.62)		0.43 (0.11-1.66)		0.16 (0.03-0.88)		0.25 (0.06-1.06)		0.67 (0.18-2.44)		1.55 (1.21-1.98)	

### Survival predictive effects of serum glycometabolites

**Figure 2:**
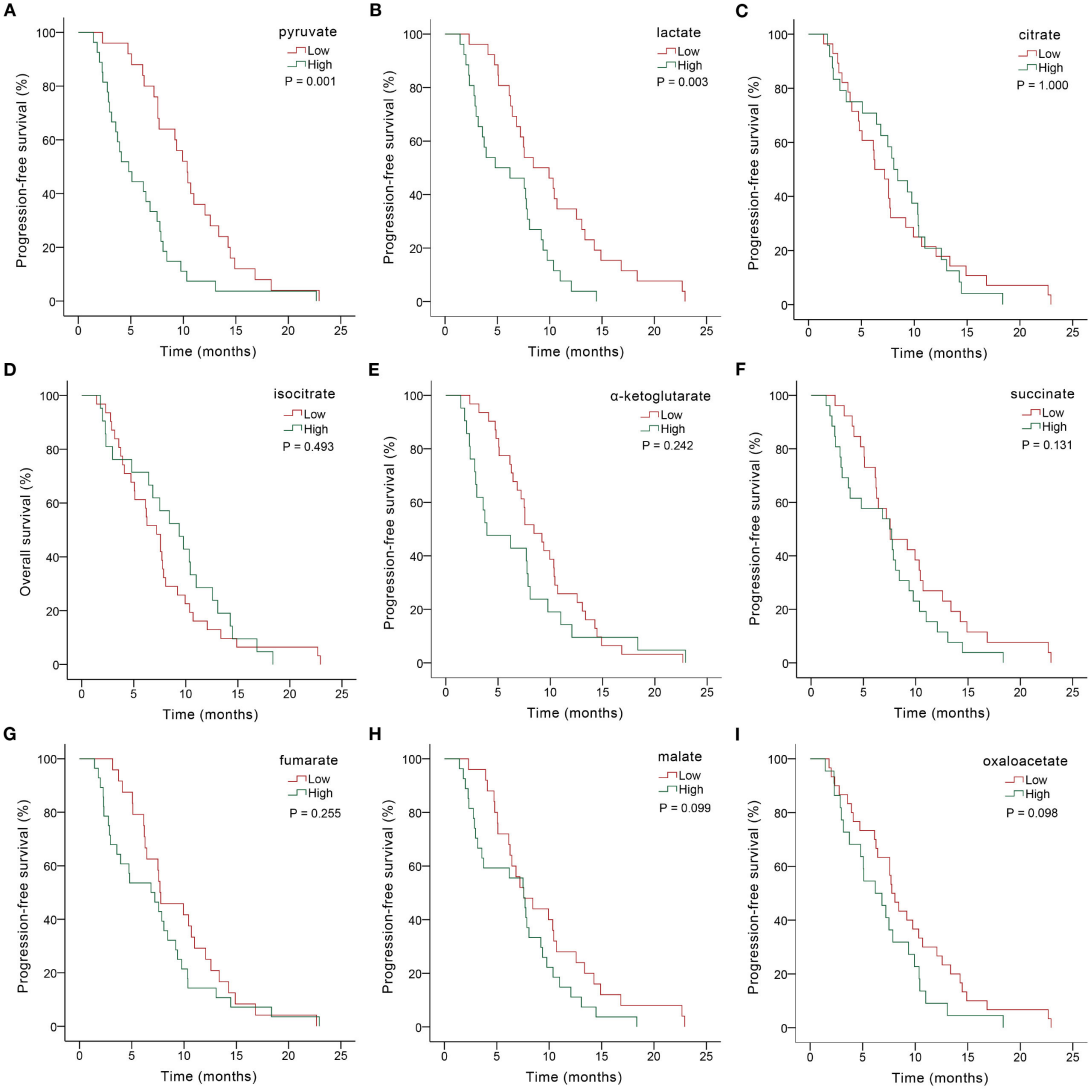
Kaplan-Meier PFS analysis based on serum glycometabolite concentrations in advanced gastric cancer patients receiving chemoimmunotherapy. Graphs depicted results for individual glycometabolites: **(A)** pyruvate, **(B)** lactate, **(C)** citrate, **(D)** isocitrate, **(E)** α-ketoglutarate, **(F)** succinate, **(G)** fumarate, **(H)** malate, **(I)** oxaloacetate.

**Figure 3:**
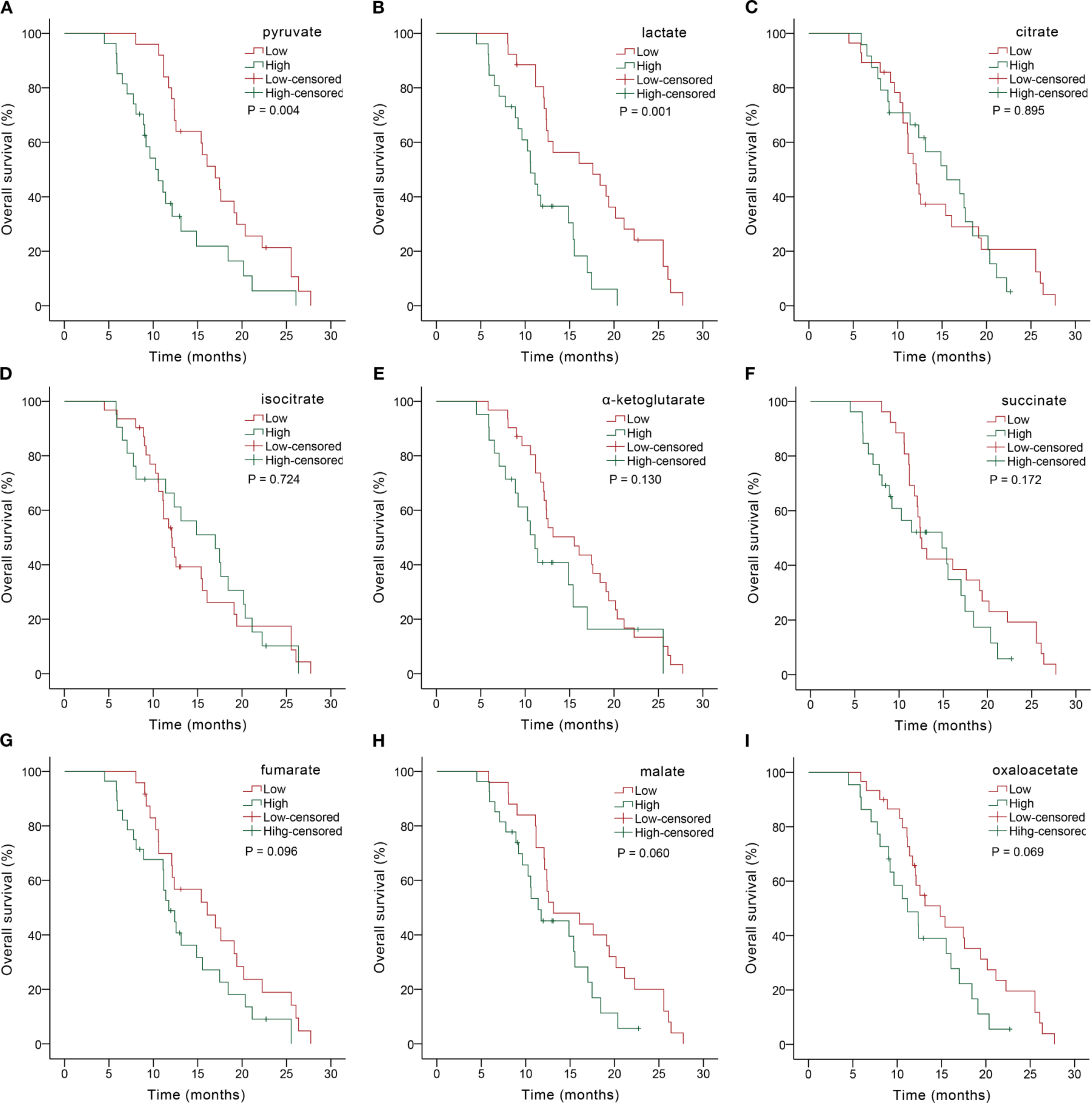
Kaplan-Meier OS analysis based on serum glycometabolite concentrations in advanced gastric cancer patients receiving chemoimmunotherapy. Graphs depicted results for individual glycometabolites: **(A)** pyruvate, **(B)** lactate, **(C)** citrate, **(D)** isocitrate, **(E)** α-ketoglutarate, **(F)** succinate, **(G)** fumarate, **(H)** malate, **(I)** oxaloacetate.

We further explored the predictive role of serum glycometabolites and found that patients in the low pyruvate (10.37 *vs.* 4.80 months, *P* = 0.001; **Figure 2A**, **Table 3**) and lactate (8.43 *vs.* 4.80 months, *P* = 0.003; **Figure 2B**, **Table 3**) concentration cohorts demonstrated significantly prolonged PFS compared to high-concentration counterparts, whereas TCA cycle metabolites showed no significant PFS associations (all *P* > 0.05, **Figures 2C-I**, **Table 3**). Patients with low concentrations of pyruvate (17.00 *vs.* 10.57 mo*nths, P* = 0.004; **Figure 3A**, **Table 3** *)*, lactate (17.60 *vs.* 10.60 months, *P* = 0.001; **Figure 3B**, **Table 3**) were associated with favorable OS, whereas TCA cycle metabolites showed no OS associations (all *P* > 0.05, **Figures 3C-I**, **Table 3**). In addition, patients with PD-L1 CPS ≥ 5 were positively associated with increased PFS (8.07 *vs.* 5.07 months, *P* = 0.004) and OS (19.40 *vs.* 11.40 months, *P* = 0.001) than those with PD-L1 CPS < 5 (**Table 3**). To further evaluate the prognostic efficacy of serum glycometabolites, we conducted time-dependent ROC analysis. Pyruvate and lactate demonstrated substantial predictive accuracy for PFS at the 6-month landmark and for OS at the 12-month landmark, respectively (all *P* < 0.05, **Table 4**).

**Table 3:** Association of serum glycometabolites with survival in advanced gastric cancer treated with chemoimmunotherapy.

Metabolite expression		PFS		OS	
		Median (95%CI)	*P* value	Median (95%CI)	*P* value
pyruvate	Low (25)	10.37 (8.623-12.11)	0.001	17.00 (14.00-20.00)	0.004
	High (27)	4.80 (2.82-6.78)		10.57 (8.35-12.78)	
lactate	Low (26)	8.43 (4.89-11.97)	0.003	17.60 (8.99-26.21)	0.001
	High (26)	4.80 (0.01-9.59)		10.60 (9.26-11.94)	
citrate	Low (28)	6.27 (4.78-7.75)	1.000	12.07 (10.44-13.70)	0.895
	High (24)	8.07 (5.83-10.31)		15.53 (10.22-20.85)	
isocitrate	Low (31)	7.20 (5.64-8.76)	0.493	12.07 (10.49-13.64)	0.724
	High (21)	9.37 (5.98-12.76)		17.00 (10.83-23.18)	
α-ketoglutarate	Low (31)	8.43 (6.40-10.47)	0.242	15.53 (10.65-20.42)	0.130
	High (21)	3.93 (0.00-7.871)		11.10 (9.49-12.72)	
succinate	Low (26)	7.57 (4.12-11.01)	0.131	12.40 (11.15-13.65)	0.172
	High (26)	7.50 (3.79-11.21)		14.87 (8.24-21.50)	
fumarate	Low (24)	7.70 (4.78-10.62)	0.255	16.07 (9.31-22.82)	0.096
	High (28)	6.83 (3.16-10.51)		11.73 (9.73-13.74)	
malate	Low (25)	7.57 (4.96-10.18)	0.099	13.13 (7.15-19.12)	0.060
	High (27)	7.57 (5.02-10.11)		11.40 (6.72-16.08)	
oxaloacetate	Low (30)	7.77 (6.60-8.93)	0.098	14.87 (10.92-18.82)	0.069
	High (22)	6.20 (3.75-8.65)		11.17 (7.18-15.15)	
PDL1 CPS	<5 (34)	5.07 (2.88-7.26)	0.004	11.40 (10.25-12.55)	0.001
	≥5 (18)	8.07 (6.89-9.25)		19.40 (16.24-22.56)	

**Table 4:** Survival predictive accuracy of serum glycometabolites via time-dependent ROC analysis in advanced gastric cancer treated with chemoimmunotherapy.

Metabolites	AUC (95%CI)			
	PFS (6 months)	*P* value	OS (12 months)	*P* value
pyruvate	0.80 (0.67-0.93)	<0.001	0.72 (0.57-0.86)	0.007
lactate	0.73 (0.60-0.87)	0.007	0.74 (0.59-0.88)	0.004
citrate	0.49 (0.31-0.66)	0.892	0.52 (0.35-0.68)	0.854
isocitrate	0.54 (0.37-0.71)	0.631	0.50 (0.34-0.66)	0.971
α-ketoglutarate	0.67 (0.52-0.83)	0.041	0.69 (0.54-0.84)	0.021
succinate	0.60 (0.42-0.78)	0.248	0.61 (0.45-0.77)	0.163
fumarate	0.73 (0.58-0.89)	0.006	0.69 (0.54-0.83)	0.021
malate	0.62 (0.45-0.80)	0.149	0.71 (0.57-0.86)	0.008
oxaloacetate	0.55 (0.38-0.73)	0.538	0.54 (0.38-0.70)	0.660

### Univariate and multivariate analyses

Univariate analysis revealed significant associations with PFS for PD-L1 CPS (*P* = 0.005), pyruvate (*P* = 0.001), and lactate (*P* = 0.004), further demonstrating robust associations with OS for PD-L1 CPS (*P* = 0.005), pyruvate (*P* = 0.006), and lactate (*P* = 0.001) (**Tables 5**, **6**). Subsequent multivariate Cox regression confirmed PD-L1 CPS ≥ 5, low pyruvate levels, and low lactate levels as independent prognostic factors for prolonged PFS and OS in advanced gastric cancer patients underwent first-line chemoimmunotherapy (all *P* < 0.05, **Tables 5**, **6**).

**Table 5:** Univariate and multivariate analyses of glycometabolites associated with PFS in advanced gastric cancer treated with chemoimmunotherapy.

Variables	Univariate		Multivariate	
	HR (95%CI)	*P* value	HR (95%CI)	*P* value
Age (≥65 *vs.* <65, year)	1.45 (0.82-2.56)	0.198		
Gender (Female *vs.* Male)	1.27 (0.59-2.72)	0.543		
G stage (G3 *vs.* G1-2)	1.71(0.82-3.55)	0.150		
HER-2 (1-2 *vs.* 0)	0.38(0.450-1.35)	0.780		
PD-L1 CPS (high *vs.* low)	0.42 (0.22-0.77)	0.005	0.30 (0.15-0.59)	<0.001
pyruvate (high *vs.* low)	2.57 (1.45-4.55)	0.001	3.79 (2.01-7.15)	<0.001
lactate (high *vs.* low)	2.43 (1.34-4.40)	0.004	2.02 (1.08-3.78)	0.028
citrate (high *vs.* low)	1.00 (0.57-1.75)	1.000		
isocitrate (high *vs.* low)	0.82 (0.47-1.45)	0.494		
α-ketoglutarate (high *vs.* low)	1.40 (0.79-2.48)	0.224		
succinate (high *vs.* low)	1.54 (0.88-2.69)	0.134		
fumarate (high *vs.* low)	1.38 (0.79-2.40)	0.257		
malate (high *vs.* low)	1.60 (0.91-2.81)	0.102		
oxaloacetate (high *vs.* low)	1.61 (0.91-2.84)	0.101		

**Table 6:** Univariate and multivariate analyses of glycometabolites associated with OS in advanced gastric cancer treated with chemoimmunotherapy.

Variables	Univariate		Multivariate	
	HR (95%CI)	*P* value	HR (95%CI)	*P* value
Age (≥65 *vs.* <65, year)	1.78 (0.97-3.27)	0.061		
Gender (Female *vs.* Male)	1.89 (0.85-4.23)	0.120		
G stage (G3 *vs.* G1-2)	1.35 (0.62-2.92)	0.449		
HER-2 (1-2 *vs.* 0)	0.68(0.38-1.22)	0.194		
PD-L1 CPS (high *vs.* low)	0.42 (0.22-0.77)	0.005	0.26 (0.12-0.58)	0.001
pyruvate (high *vs.* low)	2.34 (1.28-4.29)	0.006	3.49 (1.75-6.95)	<0.001
lactate (high *vs.* low)	3.13 (1.59-6.14)	0.001	2.24 (1.10-4.55)	0.026
citrate (high *vs.* low)	1.04 (0.56-1.93)	0.896		
isocitrate (high *vs.* low)	0.90 (0.49-1.64)	0.726		
α-ketoglutarate (high *vs.* low)	1.61 (0.86-3.03)	0.136		
succinate (high *vs.* low)	1.54 (0.83-2.85)	0.176		
fumarate (high *vs.* low)	1.67 (0.16-3.07)	0.101		
malate (high *vs.* low)	1.82 (0.97-3.43)	0.063		
oxaloacetate (high *vs.* low)	1.77 (0.95-3.30)	0.073		

## Discussion

Immunotherapy offers significant survival improvements for patients with advanced gastric cancer (3). Multiple large-scale phase III randomized controlled trials, including KEYNOTE-859, CheckMate-649, ATTRACTION-4, ORIENT-16, and RATIONALE-305, have demonstrated that combining immunotherapy with chemotherapy significantly improves the ORR and prolongs PFS and OS in patients with previously untreated HER2-negative advanced gastric cancer (14–18). However, due to treatment resistance in a subset of patients, it is crucial to meticulously select appropriate patients to ensure therapeutic efficacy. In the present study, we evaluated the predictive significance of serum glycometabolites in patients undergoing first-line chemoimmunotherapy for advanced gastric cancer, and revealed that elevated serum glycolytic metabolites (lactate and pyruvate) were significantly associated with reduced DCR, and served as independent prognostic biomarkers for predicting shorter PFS and OS.

Glycolysis encompasses the catabolic breakdown of glucose or glycogen into pyruvate, yielding modest amounts of ATP. Notably, even under aerobic conditions, the pyruvate produced by glycolysis is reduced to lactate through lactate dehydrogenase catalysis (10). Moreover, tumor cells tend to utilize glycolysis to promote cell proliferation and migration, and to evade immune surveillance by utilizing the glycolytic metabolites (19). Pyruvate plays a central role in glycolysis and is critical for tumor growth; it is also associated with the progression of lung cancer (20). As a pivotal TME regulator, lactate drives immunosuppression mainly by impairing functions of cytotoxic T-cells, blocking antigen presentation of dendritic cells, polarizing M2 macrophages, suppressing activity of NK cells, and amplifying suppression of Treg cells (21–25). Numerous studies demonstrate that overexpression of glycolysis-related genes associates with poor prognosis and immunotherapy response across diverse malignancies (26–30). Excessive lactate accumulation within the TME induces lactic acidosis in patients, with hyperlactatemia (>2 mmol/L) associated with high tumor burden and elevated long-term mortality in lymphoma cohorts (10). Previous study detected elevated serum pyruvate and lactate levels in immunotherapy non-responders of lung cancer (31). Our data corroborate this association, with significantly higher serum levels of pyruvate and lactate associated with reduced DCR in gastric cancer. Meanwhile, Mei et al. (32) have identified a significant inverse association between serum pyruvate and survival outcomes (PFS, and OS) in advanced non-small cell lung cancer patients receiving chemoimmunotherapy. Mirroring this phenomenon in advanced gastric cancer, our prognostic analysis confirmed that elevated pyruvate levels independently predict inferior PFS and OS. Further analysis established elevated lactate as an independent predictor of shortened survival in immunotherapy-treated patients with advanced gastric cancer.

Cancer cells retain functional oxidative phosphorylation in addition to the aerobic glycolysis pathway. Certain tumors even use oxidative phosphorylation as their primary ATP production mechanism (33). TCA metabolites, such as citrate, α-ketoglutarate, succinate, fumarate, regulate multiple facets of cancer progression (34). Certain metabolites have cytokine-like effects in immune cells, exerting both pro-inflammatory and anti-inflammatory functions. However, the TME conditions redirect TCA cycle metabolites toward predominantly pro-tumorigenic functions (9). For example, α-ketoglutarate promotes the M2 polarization of macrophages and inhibits their antigen presentation, thus promoting immune evasion (35, 36). Tumoral succinate drives macrophage M2 polarization to promote tumor metastasis, while microbiota-derived succinate impairs CD8^+^ T cell immunity, reducing anti-PD-1 efficacy (37–39). Similarly, cancer-derived fumarate suppresses CD8^+^ T cell anti-tumor function, yet its therapeutic depletion enhances CAR-T cell anti-tumor efficacy (40); in parallel, fumarate inhibits B cell activation, proliferation, and inflammatory responses (41). Beyond the observed inverse associations of α-ketoglutarate, and fumarate with DCR, subsequent analysis revealed no significant associations between any TCA cycle metabolites and clinical outcomes, including OS, PFS, or therapeutic efficacy in this study. The lack of predictive significance of these metabolites may be attributed to the complexity of the TCA cycle metabolic network and the dynamic variability of the metabolites (42).

Liquid biopsy is being increasingly utilized for molecular profiling of cancers, thereby enabling precision oncology approaches (6). Serum biomarkers, including circulating tumor DNA, exosomes, microRNAs, and metabolites, can reflect tumor characteristics and treatment responses (43, 44). Our study redirected biomarker discovery from tissue-based paradigms to serum metabolites directly resulting from tumor metabolic reprogramming (4, 45). The results demonstrated that elevated serum lactate and pyruvate levels serve as independent prognostic biomarkers strongly associated with therapeutic efficacy, exhibiting predictive efficacy comparable to PD-L1 CPS. These findings advance precise stratification of gastric cancer patients and personalized immunotherapy regimens. Additionally, the identification of metabolic biomarkers establishes a method that can be longitudinally monitored for treatment outcomes, enabling timely adjustments in clinical management (46). Notably, serum lactate and pyruvate offer a non-invasive liquid biopsy approach. Their utility for early diagnosis of gastric cancer, disease progression assessment, and monitoring diverse anti-tumor therapies warrants further investigation.

Several limitations warrant acknowledgment as they may affect the interpretation and generalizability of our findings. First, limited statistical power stemming from the modest cohort size and constrained follow-up duration may compromise the robustness of our conclusions. Second, the lack of serial metabolite measurements prevents analysis of dynamic expression changes, which may compromise result validity and undermine inference robustness, necessitating future longitudinal studies to address this limitation. Third, the absence of a healthy control cohort precludes direct comparison of serum metabolite concentrations between cancer patients and healthy individuals, thereby preventing establishment of optimal clinical cut-off values for diagnostic and/or prognostic stratification.

In conclusion, this study demonstrates that elevated serum lactate and pyruvate—novel glycolytic biomarkers—are associated with reduced DCR, shorter PFS, and inferior OS in patients with advanced gastric cancer receiving chemoimmunotherapy, thereby offering promise for personalizing therapeutic strategies and monitoring treatment efficacy. Future research should prioritize validating the clinical significance of these biomarkers in gastric cancer and promote their application across other gastrointestinal malignancies, while also integrating them with imaging, genomics, or immunoassays to guide precision oncology therapeutics.

## Data Availability

The raw data supporting the conclusions of this article will be made available by the authors, without undue reservation.

## References

[1] Bray F Laversanne M Sung H Ferlay J Siegel RL Soerjomataram I. Global cancer statistics 2022: GLOBOCAN estimates of incidence and mortality worldwide for 36 cancers in 185 countries. CA Cancer J Clin. (2024) 74:229–63. doi: 10.3322/caac.21834, PMID: 38572751

[2] Sundar R Nakayama I Markar SR Shitara K van Laarhoven H Janjigian YY. Gastric cancer. Lancet. (2025) 405:2087–102. doi: 10.1016/S0140-6736(25)00052-2, PMID: 40319897

[3] Eom SS Ryu KW Han HS Kong SH. A comprehensive and comparative review of global gastric cancer treatment guidelines: 2024 update. J Gastric Cancer. (2025) 25:153–76. doi: 10.5230/jgc.2025.25.e10, PMID: 39822173 PMC11739642

[4] Sun F Gao X Wang W Zhao X Zhang J Zhu Y. Predictive biomarkers in the era of immunotherapy for gastric cancer: current achievements and future perspectives. Front Immunol. (2025) 16:1599908. doi: 10.3389/fimmu.2025.1599908, PMID: 40438098 PMC12116377

[5] Röcken C. Predictive biomarkers in gastric cancer. J Cancer Res Clin Oncol. (2023) 149:467–81. doi: 10.1007/s00432-022-04408-0, PMID: 36260159 PMC9889517

[6] Ma S Zhou M Xu Y Gu X Zou M Abudushalamu G. Clinical application and detection techniques of liquid biopsy in gastric cancer. Mol Cancer. (2023) 22:7. doi: 10.1186/s12943-023-01715-z, PMID: 36627698 PMC9832643

[7] Ye L Jiang Y Zhang M. Crosstalk between glucose metabolism, lactate production and immune response modulation. Cytokine Growth Factor Rev. (2022) 68:81–92. doi: 10.1016/j.cytogfr.2022.11.001, PMID: 36376165

[8] Shang Z Ma Z Wu E Chen X Tuo B Li T. Effect of metabolic reprogramming on the immune microenvironment in gastric cancer. BioMed Pharmacother. (2024) 170:116030. doi: 10.1016/j.biopha.2023.116030, PMID: 38128177

[9] Zasłona Z O’Neill L. Cytokine-like roles for metabolites in immunity. Mol Cell. (2020) 78:814–23. doi: 10.1016/j.molcel.2020.04.002, PMID: 32333837

[10] Kooshan Z Cárdenas-Piedra L Clements J Batra J. Glycolysis, the sweet appetite of the tumor microenvironment. Cancer Lett. (2024) 600:217156. doi: 10.1016/j.canlet.2024.217156, PMID: 39127341

[11] Kubik J Humeniuk E Adamczuk G Madej-Czerwonka B Korga-Plewko A. Targeting energy metabolism in cancer treatment. Int J Mol Sci. (2022) 23. doi: 10.3390/ijms23105572, PMID: 35628385 PMC9146201

[12] Chakraborty S Sharma G Karmakar S Banerjee S. Multi-OMICS approaches in cancer biology: New era in cancer therapy. Biochim Biophys Acta Mol Basis Dis. (2024) 1870:167120. doi: 10.1016/j.bbadis.2024.167120, PMID: 38484941

[13] Donisi C Pretta A Pusceddu V Ziranu P Lai E Puzzoni M. Immunotherapy and cancer: the multi-omics perspective. Int J Mol Sci. (2024) 25. doi: 10.3390/ijms25063563, PMID: 38542536 PMC10971308

[14] Rha SY Oh DY Yañez P Bai Y Ryu MH Lee J. Pembrolizumab plus chemotherapy versus placebo plus chemotherapy for HER2-negative advanced gastric cancer (KEYNOTE-859): a multicentre, randomised, double-blind, phase 3 trial. Lancet Oncol. (2023) 24:1181–95. doi: 10.1016/S1470-2045(23)00515-6, PMID: 37875143

[15] Janjigian YY Shitara K Moehler M Garrido M Salman P Shen L. First-line nivolumab plus chemotherapy versus chemotherapy alone for advanced gastric, gastro-oesophageal junction, and oesophageal adenocarcinoma (CheckMate 649): a randomised, open-label, phase 3 trial. Lancet. (2021) 398:27–40. doi: 10.1016/S0140-6736(21)00797-2, PMID: 34102137 PMC8436782

[16] Kang YK Chen LT Ryu MH Oh DY Oh SC Chung HC. Nivolumab plus chemotherapy versus placebo plus chemotherapy in patients with HER2-negative, untreated, unresectable advanced or recurrent gastric or gastro-oesophageal junction cancer (ATTRACTION-4): a randomised, multicentre, double-blind, placebo-controlled, phase 3 trial. Lancet Oncol. (2022) 23:234–47. doi: 10.1016/S1470-2045(21)00692-6, PMID: 35030335

[17] Xu J Jiang H Pan Y Gu K Cang S Han L. Sintilimab plus chemotherapy for unresectable gastric or gastroesophageal junction cancer: the ORIENT-16 randomized clinical trial. JAMA. (2023) 330:2064–74. doi: 10.1001/jama.2023.19918, PMID: 38051328 PMC10698618

[18] Qiu MZ Oh DY Kato K Arkenau T Tabernero J Correa MC. Tislelizumab plus chemotherapy versus placebo plus chemotherapy as first line treatment for advanced gastric or gastro-oesophageal junction adenocarcinoma: RATIONALE-305 randomised, double blind, phase 3 trial. BMJ. (2024) 385:e078876. doi: 10.1136/bmj-2023-078876, PMID: 38806195

[19] Arner EN Rathmell JC. Metabolic programming and immune suppression in the tumor microenvironment. Cancer Cell. (2023) 41:421–33. doi: 10.1016/j.ccell.2023.01.009, PMID: 36801000 PMC10023409

[20] Sellers K Fox MP Bousamra M 2nd Slone SP Higashi RM Miller DM. Pyruvate carboxylase is critical for non-small-cell lung cancer proliferation. J Clin Invest. (2015) 125:687–98. doi: 10.1172/JCI72873, PMID: 25607840 PMC4319441

[21] Fischer K Hoffmann P Voelkl S Meidenbauer N Ammer J Edinger M. Inhibitory effect of tumor cell-derived lactic acid on human T cells. Blood. (2007) 109:3812–9. doi: 10.1182/blood-2006-07-035972, PMID: 17255361

[22] Gottfried E Kunz-Schughart LA Ebner S Mueller-Klieser W Hoves S Andreesen R. Tumor-derived lactic acid modulates dendritic cell activation and antigen expression. Blood. (2006) 107:2013–21. doi: 10.1182/blood-2005-05-1795, PMID: 16278308

[23] Selleri S Bifsha P Civini S Pacelli C Dieng MM Lemieux W. Human mesenchymal stromal cell-secreted lactate induces M2-macrophage differentiation by metabolic reprogramming. Oncotarget. (2016) 7:30193–210. doi: 10.18632/oncotarget.8623, PMID: 27070086 PMC5058674

[24] Brand A Singer K Koehl GE Kolitzus M Schoenhammer G Thiel A. LDHA-associated lactic acid production blunts tumor immunosurveillance by T and NK cells. Cell Metab. (2016) 24:657–71. doi: 10.1016/j.cmet.2016.08.011, PMID: 27641098

[25] Gerriets VA Kishton RJ Johnson MO Cohen S Siska PJ Nichols AG. Foxp3 and Toll-like receptor signaling balance T(reg) cell anabolic metabolism for suppression. Nat Immunol. (2016) 17:1459–66. doi: 10.1038/ni.3577, PMID: 27695003 PMC5215903

[26] Zeng L Liang L Fang X Xiang S Dai C Zheng T. Glycolysis induces Th2 cell infiltration and significantly affects prognosis and immunotherapy response to lung adenocarcinoma. Funct Integr Genomics. (2023) 23:221. doi: 10.1007/s10142-023-01155-4, PMID: 37400733

[27] Xu L Liu J An Y Zhou L Sun H Xu Z. Glycolysis-related genes predict prognosis and indicate immune microenvironment features in gastric cancer. BMC Cancer. (2024) 24:979. doi: 10.1186/s12885-024-12747-z, PMID: 39118022 PMC11313097

[28] Xu Q Miao D Song X Chen Z Zeng L Zhao L. Glycolysis-related gene signature can predict survival and immune status of hepatocellular carcinoma. Ann Surg Oncol. (2022) 29:3963–76. doi: 10.1245/s10434-022-11502-7, PMID: 35266081

[29] Shen C Suo Y Guo J Su W Zhang Z Yang S. Development and validation of a glycolysis-associated gene signature for predicting the prognosis, immune landscape, and drug sensitivity in bladder cancer. Front Immunol. (2024) 15:1430583. doi: 10.3389/fimmu.2024.1430583, PMID: 39867879 PMC11757262

[30] Cui Z Sun G Bhandari R Lu J Zhang M Bhandari R. Comprehensive analysis of glycolysis-related genes for prognosis, immune features, and candidate drug development in colon cancer. Front Cell Dev Biol. (2021) 9:684322. doi: 10.3389/fcell.2021.684322, PMID: 34422808 PMC8377503

[31] Ghini V Laera L Fantechi B Monte FD Benelli M McCartney A. Metabolomics to assess response to immune checkpoint inhibitors in patients with non-small-cell lung cancer. Cancers (Basel). (2020) 12:3574. doi: 10.3390/cancers12123574, PMID: 33265926 PMC7760033

[32] Mei L Zhang Z Li X Yang Y Qi R. Metabolomics profiling in prediction of chemo-immunotherapy efficiency in advanced non-small cell lung cancer. Front Oncol. (2022) 12:1025046. doi: 10.3389/fonc.2022.1025046, PMID: 36733356 PMC9887290

[33] Zong WX Rabinowitz JD White E. Mitochondria and cancer. Mol Cell. (2016) 61:667–76. doi: 10.1016/j.molcel.2016.02.011, PMID: 26942671 PMC4779192

[34] Eniafe J Jiang S. The functional roles of TCA cycle metabolites in cancer. Oncogene. (2021) 40:3351–63. doi: 10.1038/s41388-020-01639-8, PMID: 33864000

[35] Zhang N Sun L Zhou S Ji C Cui T Chu Q. Cholangiocarcinoma PDHA1 succinylation suppresses macrophage antigen presentation via alpha-ketoglutaric acid accumulation. Nat Commun. (2025) 16:3177. doi: 10.1038/s41467-025-58429-7, PMID: 40180922 PMC11968997

[36] Li M Chen Q Zhou M Li X Wang Z Wang J. α-ketoglutaric acid reprograms macrophages by altering energy metabolism to promote the regeneration of small-diameter vascular grafts. ACS Biomater Sci Eng. (2025) 11:518–30. doi: 10.1021/acsbiomaterials.4c01702, PMID: 39604080

[37] Jiang SS Xie YL Xiao XY Kang ZR Lin XL Zhang L. Fusobacterium nucleatum-derived succinic acid induces tumor resistance to immunotherapy in colorectal cancer. Cell Host Microbe. (2023) 31:781–797.e9. doi: 10.1016/j.chom.2023.04.010, PMID: 37130518

[38] Wu JY Huang TW Hsieh YT Wang YF Yen CC Lee GL. Cancer-derived succinate promotes macrophage polarization and cancer metastasis via succinate receptor. Mol Cell. (2020) 77:213–227.e5. doi: 10.1016/j.molcel.2019.10.023, PMID: 31735641

[39] Gudgeon N Munford H Bishop EL Hill J Fulton-Ward T Bending D. Succinate uptake by T cells suppresses their effector function via inhibition of mitochondrial glucose oxidation. Cell Rep. (2022) 40:111193. doi: 10.1016/j.celrep.2022.111193, PMID: 35977513 PMC9638018

[40] Cheng J Yan J Liu Y Shi J Wang H Zhou H. Cancer-cell-derived fumarate suppresses the anti-tumor capacity of CD8+ T cells in the tumor microenvironment. Cell Metab. (2023) 35:961–978.e10. doi: 10.1016/j.cmet.2023.04.017, PMID: 37178684

[41] Cheng J Xiao Y Jiang P. Fumarate integrates metabolism and immunity in diseases. Trends Endocrinol Metab. (2025). doi: 10.1016/j.tem.2025.03.008, PMID: 40246619

[42] Corbet C Feron O. Cancer cell metabolism and mitochondria: Nutrient plasticity for TCA cycle fueling. Biochim Biophys Acta Rev Cancer. (2017) 1868:7–15. doi: 10.1016/j.bbcan.2017.01.002, PMID: 28110019

[43] You W Shang B Sun J Liu X Su L Jiang S. Mechanistic insight of predictive biomarkers for antitumor PD−1/PD−L1 blockade: A paradigm shift towards immunome evaluation (Review). Oncol Rep. (2020) 44:424–37. doi: 10.3892/or.2020.7643, PMID: 32627031 PMC7336519

[44] Jiang T Mei L Yang X Sun T Wang Z Ji Y. Biomarkers of gastric cancer: current advancement. Heliyon. (2022) 8:e10899. doi: 10.1016/j.heliyon.2022.e10899, PMID: 36247151 PMC9561735

[45] Faubert B Solmonson A DeBerardinis RJ. Metabolic reprogramming and cancer progression. Science. (2020) 368:eaaw5473. doi: 10.1126/science.aaw5473, PMID: 32273439 PMC7227780

[46] Rayamajhi S Sipes J Tetlow AL Saha S Bansal A Godwin AK. Extracellular vesicles as liquid biopsy biomarkers across the cancer journey: from early detection to recurrence. Clin Chem. (2024) 70:206–19. doi: 10.1093/clinchem/hvad176, PMID: 38175602 PMC12374260

